# Oesophagopleural fistula after pneumonectomy: A systematic review and case series

**DOI:** 10.1308/rcsann.2023.0053

**Published:** 2023-08-29

**Authors:** L Phelan, GR Layton, EH Lee, J Halle-Smith, E Bishay, EA Griffiths

**Affiliations:** ^1^ University Hospitals Birmingham NHS Foundation Trust, UK; ^2^ University Hospitals of Leicester NHS Trust, UK; ^3^ University of Birmingham Medical School, UK; ^4^ Institute of Cancer and Genomic Sciences, College of Medical and Dental Sciences, University of Birmingham, UK

**Keywords:** Pneumonectomy, Fistula, Empyema, Pleura

## Abstract

**Introduction:**

There is a paucity of data on the optimal management of oesophagopleural fistula (OPF) following pneumonectomy. The current published literature is limited to case reports and small case series. Although rare, OPF can have a significant impact on both the morbidity and mortality of patients.

**Methods:**

Two cases of OPF managed at our institution were reported. A systematic review was then conducted in line with Preferred Reporting Items for Systematic Reviews and Meta-Analyses guidance concerning OPF following pneumonectomy. Demographic, operative and management data were analysed.

**Findings:**

Systematic review-identified data pertaining to 59 patients from 31 papers was collated. Median patient age was 59.5 years with pneumonectomy performed typically for malignancy (68%) or tuberculosis (19%). Median time from pneumonectomy to a diagnosis of OPF was 12.5 months. Twenty-five per cent of the patients had a synchronous bronchopleural fistula. Management of OPF in this setting is heterogenous. Conservative management was often reserved for asymptomatic or unfit patients. The remainder underwent endoscopic or surgical correction of the fistulae or a combination of the two with varying outcomes. Median follow-up was 18 months. All-cause mortality was 31% (18/59) with a median duration from pneumonectomy to death of 35 days (range 1–1,095).

**Conclusions:**

Major heterogeneity of management for this rare complication hinders the introduction of standardised guidance of post-pneumonectomy OPF. Surgical and endoscopic intervention is feasible and can be successful in specialist centres. Adopting an multidisciplinary team approach involving both oesophagogastric and thoracic surgery teams and the introduction of a registry database of postoperative complications are likely to yield optimal outcomes.

## Introduction

Pneumonectomy is considered a high-risk procedure, although a necessary and effective treatment option for selected patients with advanced lung cancer.^1^ Oesophagopleural fistula (OPF) is a pathological connection between the oesophagus and pleural space resulting in recurrent infection and systemic compromise, and is a highly morbid complication of pneumonectomy occurring in up to 1% of patients, with a mortality rate of 49%–63%.^2–7^ Although rates of pneumonectomy are declining, awareness of the prevention and treatment of postoperative complications, including OPF, is essential for thoracic and oesophagogastric surgeons.

Literature on OPF post pneumonectomy is limited to case reports or small cases series, with no systematic reviews or higher levels of evidence. As such, practice is varied, with no international guidelines or consensus on the optimal management for patients with OPF post pneumonectomy. There remains equipoise regarding the gold standard of treatment^7^ to provide the best quality of life, lowest mortality and highest rates of fistulae resolution.

We describe the treatment offered to two patients who presented with an OPF post pneumonectomy and provide the first systematic review of OPF following pneumonectomy.

## Case histories

### Patient 1

A 76-year-old Caucasian man presented 8 years after pneumonectomy with several weeks of progressive shortness of breath at rest associated with orthopnoea and a 15% weight loss over the preceding 4 months.

He previously had a poorly differentiated squamous cell carcinoma and large cell neuroendocrine tumour of the lung for which he underwent curative left intrapericardial pneumonectomy in 2011 and was discharged 6 days postoperatively. The left lower lobe of the lung was adherent to the chest wall, mediastinum and diaphragm, and was mobilised extrapleurally as a result. Staging was pT2bN1M0 and he had four cycles of adjuvant chemotherapy before being discharged from follow-up after 5 years post pneumonectomy.

His other past medical history included an abdominal aortic aneurysm, myocardial infarct, congestive cardiac failure, chronic obstructive pulmonary disease and 30-pack a year smoking history.

On examination, there was reduced air entry and dullness to percussion throughout the lower left hemithorax. Heart sounds were normal. Jugular venous pressure was not elevated and there was no pedal oedema. Admission blood tests showed raised inflammatory markers (c-reactive protein 197mg/l (0–5), white cell count 6.9 × 10^9^/l (4.0–11.0)), a microcytic anaemia (haemoglobin 97g/l (135–180), mean corpuscular volume 79.3fl (80.0–99.0)) and raised D-Dimer and hypoalbuminaemia (D Dimer 658ng/ml (0–250) and albumin 19g/l (35–50)).

**Figure 1:**
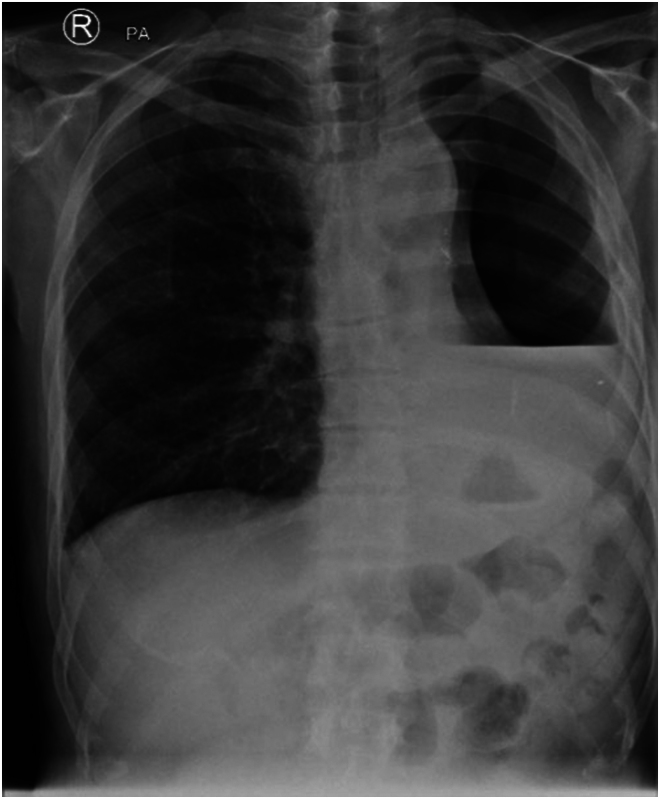
Left hydropneumothorax as identified on admission chest X-ray

**Figure 2:**
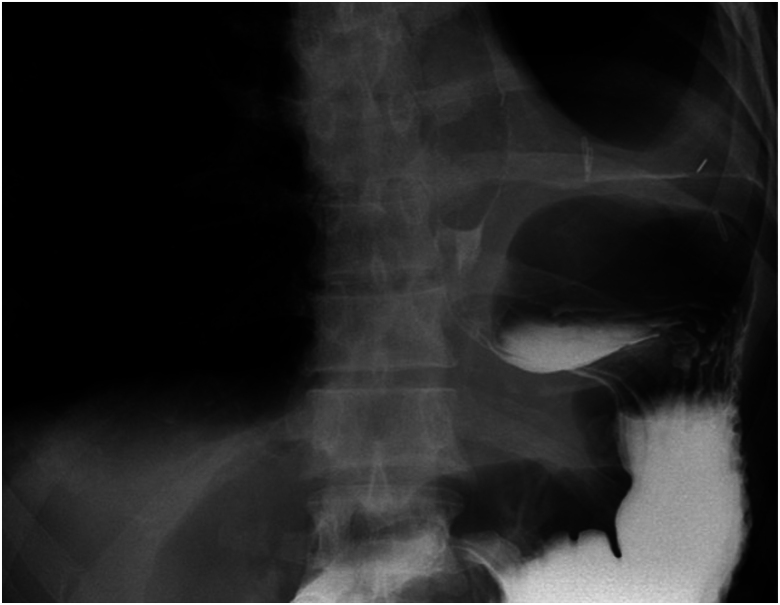
Water-soluble contrast swallow demonstrating oesophagopleural fistula between the distal oesophagus and left pleural cavity

A chest x-ray on admission (Figure 1) demonstrated left hydropneumothorax. Sequential computed tomography (CT) imaging of the thorax raised the possibility of an OPF, which was subsequently confirmed a water-soluble contrast swallow (Figure 2). An ultrasound-guided Seldinger chest drain was inserted and immediately drained pus, which when cultured isolated *Staphylococcus epidermidis*, *Escherichia coli* and *Candida parapsilosis.* Empyema was treated with antibiotics and antifungals with daily drain flushes.

Once his initial condition improved, oesophagogastroduodenoscopy (OGD) identified the fistula at 34cm in the left middle oesophagus and a covered oesophageal (ELLA 85mm) stent was inserted. Unfortunately, the stent migrated and was removed, with the patient being made nil by mouth and a jejunostomy inserted for nutrition. Because of ileus the patient required total parenteral nutrition.

**Figure 3:**
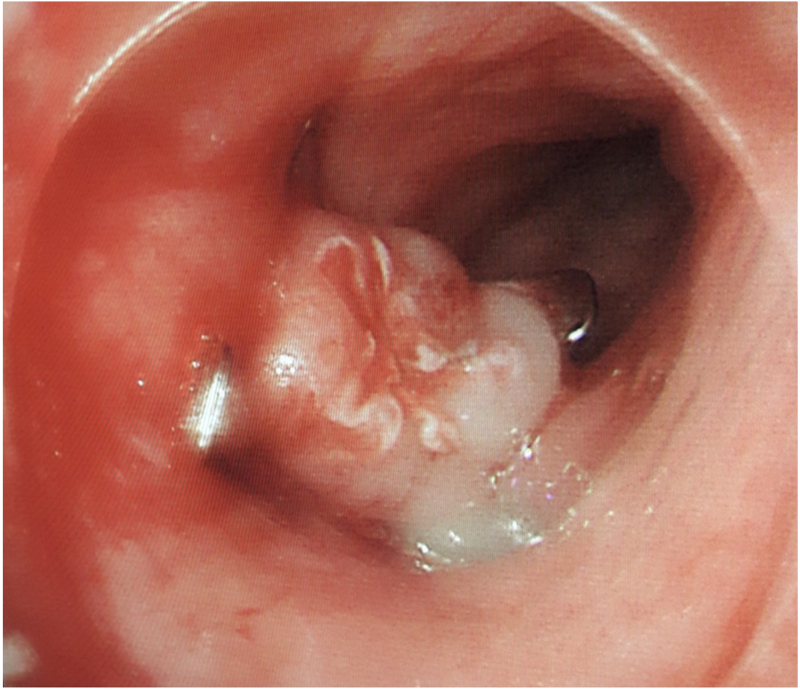
Over the scope clip to close oesophagopleural fistula

Repeat water-soluble contrast swallow a month following initial presentation suggested resolution of the OPF, although intercostal drain outputs remained suggestive of a persistent fistula. A tubogram via a nasogastric (NG) tube confirmed ongoing enterothoracic leak. Another OGD was performed, and the internal opening was targeted with argon beam diathermy and clipped with an over the scope clip (OTSC) (Figure 3). Following this, a further contrast swallow failed to identify any ongoing OPF; however, the drain output had failed to decrease, and a CT scan confirmed worsening left pleura thickening and an increasing left hydrothorax.

**Figure 5:**
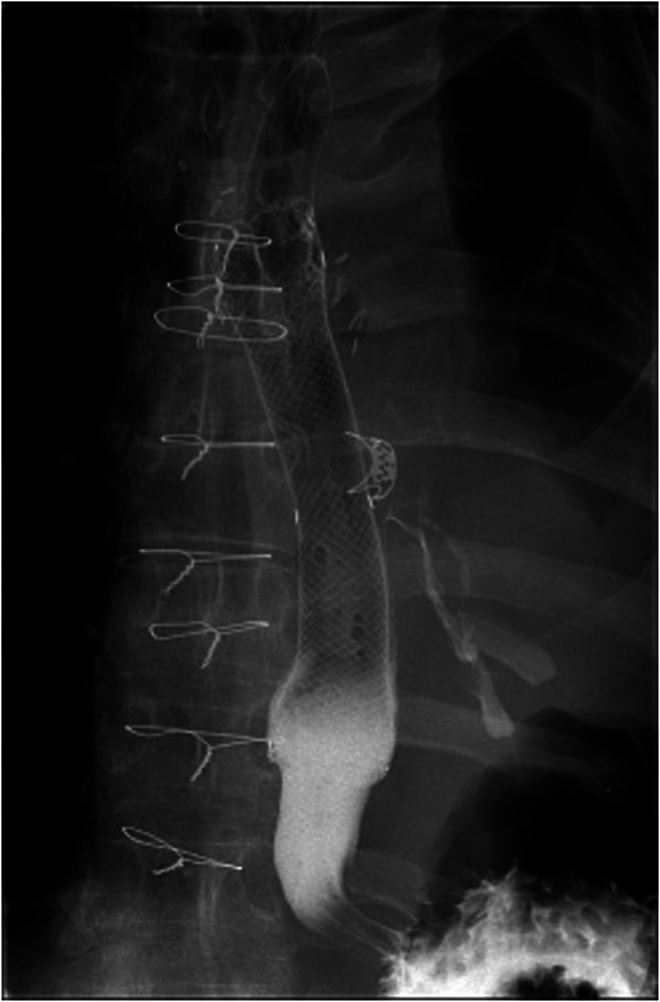
Water-soluble contrast swallow following over the scope clip and ELLA stent insertion

Because the fistula was not healing, the patient was transferred to the tertiary thoracic surgery unit. Control of chest sepsis was achieved with placement of a second drain and regular irrigation with both normal saline and antibiotics. Clinically the patient improved, although the drain fluid cultures were positive for *Pseudomonas aeruginosa* and *Enterococcus faecium*. The apical drain was removed, and he was discharged home 85 days after the initial admission with the basal drain in situ with a flutter bag on long-term ciprofloxacin.

The patient remained under close follow-up in the thoracic surgery outpatient department and had regular drain samples sent to microbiology for analysis. Four months following discharge, chest drain cultures grew *Pseudomonas aeruginosa* and *Corynebacterium striatum* with the *Pseudomonas* species being resistant to multiple antibiotics including ciprofloxacin. It was believed that these may represent contaminants from a biofilm in the drain and given that the patient was clinically well, long-term ciprofloxacin was continued and microbiology consultation was sought. Five months following discharge, the drainage bag connection fell off and this was re-sited at another hospital site. They used a three-way tap during this and the patient had no drain output for 36 hours, but despite this remained well. He therefore had his drain removed in the following weeks, 7 months following the initial admission. The patient was discharged from the thoracic surgery clinic, but later died under the care of the Macmillan cancer team at another hospital under unclear clinical circumstances, 10 years following pneumonectomy.

### Patient 2

**Figure 4:**
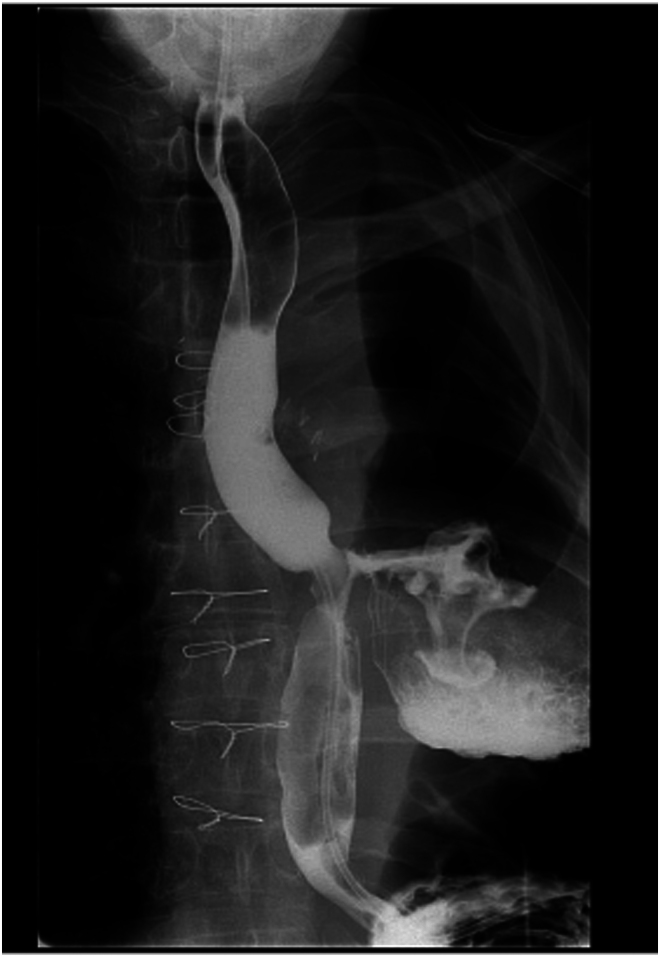
Water-soluble contrast swallow at admission in Case 2

A 53-year-old man was transferred to our tertiary oesophagogastric unit with an OPF demonstrated on barium swallow after presenting to his local hospital feeling unwell. He had previously undergone a left pneumonectomy for lung cancer via left thoracotomy (T2aN1 squamous cell carcinoma of the left upper lobe invading the left main bronchus) in June 2019, some 3.5 years before presentation. He developed a late empyema and bronchopleural fistula (BPF) in September 2019 which was treated by median sternotomy, re-stapling of left main at carina and left video-assisted thoracic surgery (VATS) wash out. Thereafter he had an uneventful follow-up with regards to his lung cancer. He was fit and well, taking no regular prescribed medications prior to admission. On transfer to our unit, the patient had a repeat water-soluble contrast swallow that demonstrated an OPF in the mid oesophagus (Figure 4). The collection in his thorax was assessed by the thoracic surgery team and was not drained because he was systemically well. He underwent an OGD to assess the fistula defect. The perforation site was 35cm from the incisors and was smaller than the 11mm gastroscope. A biopsy showed chronic oesophagitis. He had a subsequent OGD and placement of an OTSC over the fistula. This was felt to be only partially successful at the time of placement and so a repeat OGD was carried out the following day and an ELLA fully covered oesophageal stent was placed.

A repeat water-soluble contrast swallow (Figure 5) showed a persisting fistula just below the OTSC although the pleural collection had reduced in size compared with previous examinations; the fistula was demonstrated only on retrograde passage of contrast around the distal stent when the patient coughed. The naso-jejunal tube was removed, and he had a naso-gastric tube (NG) placed. The patient was tolerating a liquid diet and was discharged home with ongoing NG feeding and remains under follow-up.

**Figure 6:**
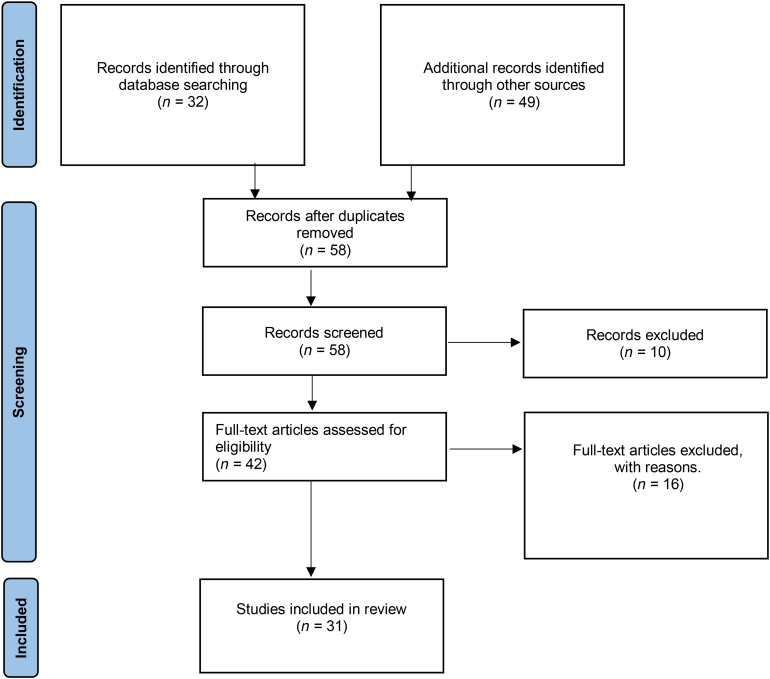
PRISMA flow diagram outlining search results. A search was conducted using the terms [(oesophago-pleural fistula) ‘OR’ (oesophagopleural fistula) ‘OR’ (esophagopleural fistula) ‘OR’ (esophago-pleural fistula) ‘OR’ (EPF) ‘OR’ (OPF) ‘AND’ [(pneumonectomy) ‘OR’ (postpneumonectomy)].

## Systematic review

### Methods

A systematic literature search was conducted in line with Preferred Reporting Items for Systematic Reviews and Meta-Analyses (PRISMA)^8^ guidance with a search of MEDLINE, Embase, Cochrane Library, BMJ Case Reports and PubMed conducted on 31 January 2023. The full search terms and a flowchart of the included studies can be found in Figure 6.

Articles published between 1969 and 2022 were eligible and were screened by two authors (LP and GRL). Abstracts were included if they were published in the English language and pertained to the presentation, diagnosis, investigation, management or outcome of OPF of any cause. The full texts and their reference lists were then screened by two authors (LP and GRL) and were included if they pertained specifically to OPF following pneumonectomy. Conflicts were resolved through discussion with senior authors (EAG and EB) to reach consensus. One paper was identified outside these search terms and although relevant to the management of OPF post pneumonectomy, was excluded because of a lack of data within the paper.^9^

### Findings

Thirty-one full texts were identified for formal review and reported information on 59 patients who had an OPF post pneumonectomy. Median patient age was 59.5 years (range 8–74) and 61% (30/49) of patients were male (gender was not specified for ten patients). Sixty-eight per cent (40/59) underwent a pneumonectomy for malignancy and 19% for tuberculosis (11/59). Stage of malignancy was reported in 12 patients and was: 1b (6/12), 2b (2/12), 3a (2/12) and 3b (2/12).

Most patients (45/59) underwent right-sided pneumonectomy; 49% of these right-sided pneumonectomy patients had an additional pericardial, oesophageal or nodal resection/intervention intraoperatively. The patient demographics are outlined alongside the details of pneumonectomy and their respective indications in Tables 1 and 2.

**Table 1:** Patient demographic and operative data

Author	Year	Country	Number of patients	Age*	Gender (male)	Histology	Original operation type	Operation side	No. of patients with previous lobe/resection on same side	No. of patients undergoing additional resection^†^
Goncalves^11^	2022	Portugal	1	64	0	B	Pneumonectomy	Right	0	0
Girelli^3^	2020	Italy	1	51	0	M	Tracheal sleeve pneumonectomy	Right	0	0
Szkorupa^27^	2018	Czech Republic	1	64	0	M	Completion pneumonectomy	Right	1	1
Noh^13^	2016	Korea	1	69	1	M	Completion pneumonectomy	Left	1	0
Kim^38^	2014	South Korea	1	55	1	M	Pneumonectomy	Right	0	0
Kim^28^	2013	Korea	1	67	1	B	Pneumonectomy	Right	0	0
Kadlec^4^	2013	UK	1	63	0	M	Pneumonectomy (after attempted left lower lobe sleeve resection (reverse sleeve))	Left	1	0
Yekeler^39^	2012	Turkey	1	NR	NR	M	Pneumonectomy	Left	0	0
Liu^29^	2006	USA	1	48	0	M	Pneumonectomy	Left	0	1
Di Franco^18^	2006	UK	1	70	1	B	Pneumonectomy	Right	0	0
Pache^5^	2005	Germany	1	64	0	B	Pneumonectomy	Right	0	0
Dosios^6^	2005	Greece	1	71	NR	M	Pneumonectomy	Right	0	1
Farivar^40^	2004	USA	1	40	0	M	Extrapleural pneumonectomy	Right	0	1
Trigui^30^	2002	France	1	60	1	M	Pneumonectomy	Right	0	0
Terzi^31^	2000	Italy	1	53	1	M	Pneumonectomy	Left	0	1
Inoue^32^	1999	Japan	1	56	1	M	Intrapericardial pneumonectomy	Right	0	1
Lauwers^21^	1996	Belgium	1	67	0	M	Pneumonectomy	Right	0	0
Massard^7^	1994	France	8	61.75*	8	7/8 M	5/8 Pneumonectomy; 2/8 completion pneumonectomy; 1/8 pleuropenumonectomy	6/8 Right	2	1
Asaoka^17^	1988	Japan	1	67	1	M	Pneumonectomy	Left	0	0
Mud^19^	1987	NR	2	45.5*	1	1/2M	2/2 Pneumonectomy	2/2 Right	0	0
Yamaguchi^15^	1989	NR	1	69	1	M	Pneumonectomy	Left	0	0
Shama^33^	1985	South Africa	7	38.14*	2	0/7 M	7/7 Pneumonectomy (1 emergency)	5/7 Right	0	1
Holdt^10^	1983	Germany	1	70	1	M	Pneumonectomy	Right	0	0
Van den Bosch^41^	1980	Netherlands	3	37.3*	2	2/3 B (1 NR)	2/3 Pneumonectomy; 1/3 completion pneumonectomy	3/3 Right	1	1
Sethi^20^	1978	USA	2	57.5*	2	2/2 M	2/2 Radical pneumonectomy	1/2 Right	0	2
Richardson^34^	1976	USA	3	58*	2	0/3 M	2/3 Pneumonectomy; 1/3 pleuropneumonectomy	2/3 Right	0	1
Efthimiadis^35^	1974	Greece	1	65	1	M	Pneumonectomy	Right	0	1
Symes^36^	1972	UK	1	62	1	M	Pneumonectomy	Left	0	0
Evans^22^	1972	UK	8	57.6*	NR	8/8 M	8/8 Pneumonectomy (6/8 had bronchial stump covered with a muscle graft)	Right	0	8
Engelman^42^	1970	NR	1	55	0	M	Radical pneumonectomy (free graft of pericardium to bronchial stump)	Right	0	1
Benjamin^37^	1969	NR	3	56*	1	3/3 M	1/3 Pneumonectomy; 2/3 Radical pneumonectomy	3/3 Right	0	3

M = malignant; B = benign; NR = not reported

*Mean age of all patients within specified study cohort

^†^Additional pericardial, oesophageal, nodal resection or intervention

**Table 2:** Summary of demographic and operative data on patients

No. of patients (range)	Age (median)	Gender	Histology	Original operation type	Operation side	No. of patients with previous lobe/resection on same side	No. of patients undergoing additional resection*
1–8	59	Male 30/59	Malignant 40/59	Pneumonectomy 38/59	Right 45/59	Yes 6/59	Yes 25/59
		Female 19/59	Benign 18/59	Pneumonectomy with muscle graft to bronchial stump 6/59	Left 14/59	No 53/59	No 34/59
		NR 10/59	NR 1/59	Radical pneumonectomy (± graft) 5/59			
				Completion pneumonectomy 5/59			
				Other 5/59			

NR = not reported

*Additional pericardial, oesophageal, nodal resection or intervention

The median time from pneumonectomy to OPF presentation was 12.5 months (range 0–600) and 15 patients (25%) had concomitant BPF. More than half of cases documented a cause or contributing causative factor (Table 3, Appendix 1 [available online]). Most frequently reported causes were chronic infection (19%), peribronchial abscess rupture into the oesophagus (14%), perforated oesophageal diverticulum (8%) and iatrogenic injury (8%).

**Table 3:** Proposed causes/contributing factors to oesophagopleural fistula formation

Cause or factor contributing to oesophagopleural fistula formation	Frequency
No cause proposed	24
Infection: chronic infection, tuberculosis, empyema	11
Peribronchial abscess that ruptured into oesophagus	8
Traction/oesophageal diverticulum (perforated)	5
Intraoperative injury	5
Local recurrence of malignancy	3
Inflammation	2
Wide mediastinal lymph node dissection	1
Focal ballooning and thinning of the oesophageal wall in the region of the operative bed	1
Ischaemic necrosis of the oesophagus in the region of oesophageal muscle resection	1
Polypropylene mesh placement after pericardial resection	1
Radiotherapy	1
Oesophageal rupture	1

### Treatment modalities

There is gross heterogeneity in the reported methods and success of treatment for these patients and patients commonly underwent multiple treatments.

#### Conservative management

Four patients underwent conservative management only with chest drain insertion and/or lavages and/or instillation of antibiotics into the chest cavity. Two fistulae resulted from peribronchial abscesses that ruptured into the oesophagus and both patients were too unwell to consider operative closure of OPF, informing the decision to manage them conservatively. These patients died at 3 and 23 days post pneumonectomy, respectively. The other two fistulae in this group resulted from local disease recurrence warranting palliative treatment only and both died within 90 days of fistula diagnosis.

#### Endoluminal approaches

Two patients underwent endoscopic management alone for OPF. One had previously been treated for an empyema secondary to BPF with chest tube drainage, antibiotics and irrigation when they developed a synchronous oesophageal fistula 36 months post pneumonectomy. Closure of the OPF was attempted with endoscopic fibrin glue injection. This failed to cease fistulation, but the patient improved clinically and was then managed conservatively. They remained well at 24 months.^10^

The second patient had an OTSC to close the fistula tract with clinical improvement.^11^

#### Surgical intervention

Forty-two patients underwent surgical treatment without any endoscopic interventions. Median time to diagnosis post pneumonectomy in this group was 14.5 months (range 0–300). Several surgical interventions were undertaken, sometimes during the same operation, and the frequency of these is summarised in Table 4 and Appendix 1 (available online)*.* Overall, 76.2% (32/42) reported treatment success. However, 12 patients died (28.6%), 7 within 90 days of pneumonectomy.

#### Combined intervention

Eight patients underwent both endoscopic and surgical interventions during their treatment for OPF (Table 4, Appendix 1 [available online]). The median time between pneumonectomy and OPF diagnosis in this group was 2.5 months (range 0–360; data not reported for one patient). All patients had successful treatment. Median follow-up time was 10 months. One patient died 372 days post intervention for OPF owing to disease progression. All other patients were well at follow-up.

**Table 4:** Summary of surgical interventions patients underwent for treatment of oesophagopleural fistulae

Operation type	Number of patients who underwent intervention during their treatment pathway
Other operation	28
Flap reconstruction ± direct closure of fistula	27
Thoracoplasty/thoracomyoplasty	17
Oesophageal reconstruction/resection	12
Primary oesophagopleural fistula closure only (no muscle/fat flap)	11
Diversion	3

Interventions directed at nutrition not included. Some patients underwent multiple interventions listed, please see Supplementary Table 2 (available online) for more information

### Outcomes

Thirty-nine patients were followed up after discharge following treatment for OPF with a median follow-up time of 18 months (range 0.5–144). Three of the 59 reported patients did not have outcomes recorded: one patient self-discharged from care following an open drainage of empyema, one declined intervention and the third was asymptomatic and therefore not offered treatment. This patient remained well at follow-up 60 months later. One patient died within 24 hours of pneumonectomy owing to gangrenous oesophagitis with a 3cm perforation.

All-cause mortality was 31% (18/59) with a median duration from pneumonectomy to death of 35 days (range 1–1,095 days).

## Discussion

OPF is a rare but important complication of pneumonectomy. This comprehensive review demonstrates that the literature is limited to case reports and small case series, and management of OPF post pneumonectomy is heterogenous. Most cases have chest drain insertion and/or irrigation of the pleural cavity as an initial management step. Endoscopic and surgical management appears to offer better outcomes, with conservative management generally reserved for patients who are asymptomatic or systemically unfit to undergo any intervention. All management is impeded by the impact of both acute and chronic sepsis and chronic malnutrition, which frequently impact these patients.^7,12,13^

Endoscopy can achieve proximal fistula control with oesophageal clipping or stents like those used for anastomotic leak following oesophagectomy and success has been demonstrated with their use for oesophagomediastinal fistulae.^6^

Stent migration, as in our case, occurs in 12% of patients and is more common when plastic stents are used compared with metallic stents.^14^ There is developing endoscopic technology that may not have been available to clinicians at the time of publication of some of the studies in this review. American Gastroenterological Association (AGA) 2021 guidelines on the endoscopic management of perforations in the gastrointestinal tract advise primary closure should be the aim in oesophageal perforation management. Perforations under 2cm can be managed using through-the-scope clips or OTSCs with clips being placed distally to proximally to ensure an optimal view.^15^ OTSCs have a reported success of 85.3%.^16^

Perforations larger than 2cm should be closed, with endoscopic suturing using self-expandable metallic stents reserved for patients in whom primary closure is not feasible allowing the defect to heal by secondary intention.^16^ To prevent migration, self-expandable metallic stents can be secured with endosuturing or OTSC placement at the proximal end.^16^ This management is consistent with our case report.

Endoscopic interventions have the benefit of being available at a wider range of sites than thoracic or oesophagogastric surgery which, in the UK, is only offered within specialised tertiary centres. However, caution is required in the use of covered oesophageal stents, and adjacent airways could potentially be compromised because of the anatomical deviation present post pneumonectomy and the close proximity of the oesophagus and central bronchial tree.^4^ Small, linear openings within the oesophagus may be amenable to endoscopic closure with endoscopic clips, although this must be avoided if ischaemia or inflammation is present.^4^ Kadlex *et al* reported the novel use of an Amplatzer atrial septal occlusion device, although this ultimately failed and was not recommended as a viable intervention for OPF post pneumonectomy.^4^ In our two cases, we used both oesophageal stents and OTSC's placed endoscopically. Although both of these ultimately did not close the fistula, the patients then improved with a period of conservative management and optimisation of nutrition throughout via both enteral and parenteral routes.

Operative interventions aim to achieve definitive treatment of fistula by direct closure, frequently with muscular reinforcement or by removing the diseased viscera completely by oesophagectomy with replacement with either gastric or colonic interposition.^4^ Reinforcement can include harvested pedicled muscle flaps, for example the intercostal bundle, or pleural flap; muscle flaps can also obliterate the cavity.^6,17–20^ Thoracoplasty is a viable alternative to obliterate the empyema^2,17,21,22^ space, but is frequently poorly tolerated and can result in chest wall deformity.^21^

When successful these aggressive surgical interventions can offer complete resolution of the fistulation, treat the chronic empyema and obviate the need for long-term chest drains. However, they are still associated with high mortality rates.^6^ Direct closure with flap reinforcement and thoracoplasty is comparable with direct suture with or without flap reinforcement alone, with a 50% cure rate.^21^ Alternative surgical options include oesophageal reconstruction with bypass or partial oesophagectomy with gastric pull-up or colonic interpositions. Again, these interventions can remove the diseased or damaged portion of oesophagus but have high mortality.^21^ Colonic interposition is an uncommonly performed operation and carries a high mortality rate.^23^ It has been recommended that operations of this kind should be centralised to higher-volume centres to try to optimise outcomes.^23^

Although in recent years the treatment of anastomotic leaks and oesophageal perforation has shown promising results with endoluminal vacuum treatment techniques, this has not been specifically reported in OPF post pneumonectomy.^24,25^ Gabryel *et al* used it successfully in a patient with a bronchial fistula and oesophageal leak after a combined lobectomy and oesophagetomy.^26^ Theoretically, it might not work in OPF alone because endoscopic vacuum treatment relies on healing of the perforation by collapse of the associated cavity with the formation of granulation tissue and then scar tissue. In the post pneumonectomy patient, the pleural cavity is empty and there is no lung tissue and minimal mediastinal tissue to allow this to occur. This adds to the complexity of treating these patients. Standard vacuum-assisted closure of the open thoracotomy wound to gain source control in combination with a wide variety of surgical techniques has been used successfully in a series of 49 complex empyema patients, which included two patients who were post pneumonectomy with OPF.^9^

We suggest a stepwise approach to management. Initial aims should be to drain and sterilise the contaminated pleural space with chest drains, Elosser flap or Clagget window. Endoscopic closure could be then attempted, although there are only small numbers reporting isolated endoscopic management in our review. Finally, surgical management can be considered, and a joint approach with upper gastrointestinal and thoracic surgeons may yield better outcomes. The timing of these procedures should be individualised depending on the circumstances.

### Study limitations

Our paper has some limitations and benefits. The current available data on management of OPF following pneumonectomy are limited to level IV evidence. This review spans many years during which there have been vast changes in cancer staging, surgical treatment and endoscopic technologies. Despite including all available data in the large time frame, we are only able to present a small number of patients. In addition, the heterogeneity of intervention makes meaningful comparison of perceived treatment ‘success’ difficult to comment upon. Finally, there is the potential for both selection and publication bias because many patients who were too unwell to undergo treatment, or studies reporting very poor outcomes and ‘treatment failure’, may not have been written up or reported. However, our systematic review represents a thorough summary of a wide range of international centres and so incorporates treatment and outcomes of patients with OPF following pneumonectomy and will be of value to surgeons who have to treat this devastating complication.

### The future

In most countries, all cardiothoracic resections are recorded in a prospectively maintained national databases such as Dendrite (UK) or the STS General Thoracic Surgery Database (US). To improve data on this subject and other complications, we suggest improved monitoring of hospital readmissions after surgery and mandatory reporting of all complications, including OPF, in patients with a history of pneumonectomy through the development of a long-term outcome and complication registry. This would allow retrospective review of a national case series for possible causative factors and any preventable measures that could be considered for future patients.

## Conclusions

Major heterogeneity in the management of this rare complication hinders the introduction of standardised guidance of post-pneumonectomy OPF. Initial management is concentrated on sepsis control, frequently utilising chest drainage, antibiotic therapy and patient optimisation with an emphasis on nutrition. Surgical and endoscopic intervention is feasible and can be successful in specialist centres. Adopting a multidisciplinary team approach involving both oesophagogastric and thoracic surgery teams, and the introduction of a registry database of postoperative complications is likely to yield optimal outcomes.

## Conflicts of interest

No conflicts exist.
